# Correction: Ali et al. Nutritional Composition and Bioactive Compounds in Tomatoes and Their Impact on Human Health and Disease: A Review. *Foods* 2021, *10*, 45

**DOI:** 10.3390/foods15030590

**Published:** 2026-02-06

**Authors:** Md Yousuf Ali, Abu Ali Ibn Sina, Shahad Saif Khandker, Lutfun Neesa, E. M. Tanvir, Alamgir Kabir, Md Ibrahim Khalil, Siew Hua Gan

**Affiliations:** 1 Laboratory of Preventive and Integrative Biomedicine, Department of Biochemistry and Molecular Biology, Jahangirnagar University, Savar, Dhaka 1342, Bangladesh; yousufbmb40ju@gmail.com (M.Y.A.); shahadsaifkhandker@gmail.com (S.S.K.); alamgirju42@gmail.com (A.K.); 2 Department of Biochemistry and Molecular Biology, Gono Bishwabidyalay, Savar, Dhaka 1344, Bangladesh; 3 Center for Personalized Nanomedicine, Australian Institute for Bioengineering and Nanotechnology (AIBN), The University of Queensland, Brisbane, QLD 4072, Australia; a.sina@uq.edu.au; 4 Department of Biotechnology and Genetic Engineering, Bangabandhu Sheikh Mujibur Rahman Science and Technology University, Gopalganj, Dhaka 8100, Bangladesh; l.neesa@bgeju.edu.bd; 5 Veterinary Drug Residue Analysis Division, Institute of Food and Radiation Biology, Atomic Energy Research Establishment, Savar, Dhaka 1349, Bangladesh; tanvir.ju.37@gmail.com; 6 School of Pharmacy, Pharmacy Australia Centre of Excellence, The University of Queensland, Woolloongabba, QLD 4102, Australia; 7 School of Pharmacy, Monash University Malaysia, Jalan Lagoon Selatan, Bandar Sunway, Selangor 47500, Malaysia

## 1. Table Legend

In the original publication [1], there was a mistake in the legends for **Table 2, Table 4, Table 7 and Table 8** as the **peel fiber, percentage of content and dry weight, dry weight, and fresh weight** were not included. The correct legend appears below. We state that the scientific conclusions are unaffected.

**Table 2.** Mineral contents in tomato peel fiber.

**Table 4.** Fatty acid contents (percentage in total fatty acid content) in tomato (dry weight).

**Table 7.** Sterol contents in tomato (mg/kg dry wt).

**Table 8.** Antioxidant constituents in tomato (fresh weight).

## 2. Error in Table

In the original publication, there was a mistake in **Table 1, Table 3, Table 4, Table 6, Table 7 and Table 8** as published. There were mistakes in **some parameters, values, and ranges.** The corrected **tables** appear below. We state that the scientific conclusions are unaffected. This correction was approved by the Academic Editor. The original publication has also been updated.

In Table 1, energy (kcal/100 g), ash (%), moisture (g/100 g), total protein (g/100 g), lipid (g/100 g), carbohydrates (g/100 g), total sugar (g/100 g), pH, total fiber values, and ranges have been corrected, and the USDA values have been added, respectively. The corrected Table 1 is as follows:

In Table 3, the unit, concentration, and range for Vitamin E, the concentration and range for Vitamin C, and the unit for folate have been corrected. The corrected Table 3 is as follows:

In Table 4, the title of the table and the heading of the second column have been corrected. The corrected Table 4 is as follows:

In Table 6, the heading of the first column, unit of each compound, concentration, and range for β-carotene and lycopene have been corrected. The corrected Table 6 is as follows:

In Table 7, the title, heading of the first column, and range for campesterol have been corrected. Additionally, in the first column, the last row “total sterol” has been added. The corrected Table 7 is as follows:

In Table 8, the title of the table, concentration, and range for Vitamin C and β-carotene have been corrected. The corrected Table 8 is as follows:

## 3. Text Correction

In Section 3.1 paragraph 3, the following correction has been made: “In a more recent study based on previously published original research articles, an average tomato consists of ash 0.6%, water 94.7 (g/100 g), moisture 93.76 (g/100 g), total protein 0.56 (g/100 g), lipid 0.13 (g/100 g), carbohydrates 5.96 (g/100 g), total sugar 4.41 (g/100 g), pH 4.38, energy 26.60 kcal/100 g, acidity 0.48%, reducing sugar 35.84%, fructose 2.88%, glucose 2.45%, sucrose 0.02% and total fiber 1.54 (g/100 g) (Table 1)”. 

In Section 3.2 paragraph 2, correction has been made as “In this review, 23 types of minerals and their amounts present in tomato peel fiber are compiled, including…”.

## Figures and Tables

**Table 1:** Proximate composition of tomato.

Parameters	Values	Range	USDA Values	References
Energy (kcal/100 g)	26.60 ± 5.56	18.00–34.67	22 Kcal/100 g	[3–5,40–52]
Ash (%)	0.60 ± 0.09	0.50–0.74	0.31 g	
Moisture (g/100 g)	93.76 ± 1.75	90.063–96.17	94.7 g/100 g	
Total protein (g/100 g)	0.56 ± 0.19	0.40–0.88	0.7 g/100 g	
Lipid (g/100 g)	0.13 ± 0.06	0.03–0.20	0.42 g/100 g	
Carbohydrates (g/100 g)	5.96 ± 1.37	3.92–8.00	3.84 g/100 g	
Total sugar (g/100 g)	4.41 ± 1.69	2.49–6.62	-	
pH	4.38 ± 0.16	4.13–4.57	-	
Acidity (%)	0.48 ± 0.07	0.39–0.55	-	
Reducing sugar (%)	35.84 ± 4.57	30.03–41.21	-	
Fructose (%)	2.88 ± 0.49	1.15–3.42	-	
Glucose (%)	2.45 ± 0.48	1.74–3.18	-	
Sucrose (%)	0.02 ± 0.05	0.01–0.02	-	
Total fiber (g/100 g)	1.54 ± 0.43	0.70–1.92	1 g/100g	

Values are expressed as mean ± standard deviation.

**Table 3:** Vitamin contents in tomato.

Vitamins	Units	Concentrations	Range	USDA Values	References
Vitamin A	IU/100 g	614.44 ± 248.18	267.33–833.00	24 µg/100 g	[4,46,61,62,65,69,73–76]
Vitamin E	mg/100 g	0.37 ± 0.15	0.17–0.62	-	
Vitamin K	μg/100 g	98.28 ± 0.00	98.28	-	
Vitamin C	mg/100 g	13.99 ± 3.73	9.03–23.80	17.8 mg/100 g	
Thiamine	mg/100 g	0.66 ± 0.44	0.04–0.98	0.056 mg/100 g	
Riboflavin	mg/100 g	0.48 ± 0.34	0.02–0.81	<0.1 mg/100 g	
Pantothenic Acid	mg/100 g	4.93 ± 0.41	4.52–5.34	-	
Vitamin B6	mg/100 g	1.51 ± 0.22	1.29–1.72	0.079 mg/100 g	
Biotin	μg/100 g	68.97 ± 0.00	68.97	0.469 µg/100 g	
Folate	μg/100 g	14.00 ± 1.00	13.00–15.00	10 µg/100 g	

Concentrations are expressed as mean ± standard deviation.

**Table 4:** Fatty acid contents (percentage in total fatty acid content) in tomato (dry weight).

Fatty Acids	Concentrations (g/100 g in Total Fatty Acid)	Range	References
Myristic acid	0.56 ± 0.22	0.32–0.93	[5,41,47,49,62]
Palmitic acid	18.07 ± 2.90	12.40–22.50	
Stearic acid	4.81 ± 1.50	2.80–6.84	
Palmitoleic acid	0.25 ± 0.10	0.03–0.32	
Oleic acid	14.24 ± 3.50	9.00–19.14	
Linoleic acid	49.40 ± 4.16	46.33–54.10	
Linolenic acid	10.17 ± 4.46	4.26–15.53	
Caproic acid	0.03 ± 0.02	0.01–0.05	
Caprylic acid	0.06 ± 0.04	0.02–0.10	
Capric acid	0.04 ± 0.03	0.01–0.07	
Heptadecanoic acid	0.26 ± 0.05	0.18–0.13	
Lauric acid	0.09 ± 0.05	0.04–0.15	
Pentadecanoic acid	0.12 ± 0.03	0.08–0.15	
Arachidic acid	0.88 ± 0.24	0.61–1.26	
Eicosadienoic acid	0.04 ± 0.02	0.02–0.06	
Arachidonic acid	0.04 ± 0.02	0.01–0.06	
Eicosapentaenoic acid	0.05 ± 0.01	0.03–0.06	
Erucic acid	0.02 ± 0.01	0.01–0.03	
Docosadienoic acid	0.07 ± 0.03	0.03–0.10	
Behenic acid	0.59 ± 0.19	0.31–0.82	
Tricosanoic acid	0.68 ± 0.54	0.16–1.52	
Lignoceric acid	0.74 ± 0.20	0.45–1.01	
Saturated fatty acid	27.40 ± 3.74	22.37–33.22	
Monounsaturated fatty acid	13.80 ± 2.42	11.00–17.66	
Polyunsaturated fatty acid	57.55 ± 23.51	55.78–58.63	
Vaccenic acid	0.53 ± 0.05	0.50–0.60	
Eicosanoic acid	0.10 ± 0.03	0.05–0.12	

Concentrations are expressed as mean ± standard deviation.

**Table 6:** Carotenoid contents in tomato.

Carotenoids	Units	Concentrations	Range	USDA Values	References
β-carotene	mg/100 g FW	0.420 ± 0.080	0.30–0.51	276 µg/100 g	[5,7,41,46,47,74,90–94]
Lycopene	mg/100 g FW	7.960 ± 1.780	5.02–9.49	2860 µg/100 g	
Lutein + zeaxanthin	μg/100 g FW	60.67 ± 43.86	18.07–123.00	56 µg/100 g	
Phytoene	μg/100 g FW	668.33 ± 361.95	430.00–1860.00	-	
Phytofluene	μg/100 g FW	500.00 ± 100.49	390.00–820.00	-	
All trans-lutein	mg/kg DW	5.00 ± 0.82	4.00–6.00	-	
All trans-β carotene	mg/kg DW	29.25 ± 27.26	4.00–75.00	-	
9-cis-β carotene	mg/kg DW	6.50 ± 2.29	3.00–9.00	-	

Concentrations are expressed as mean ± standard deviation. FW = fresh weight, DW = dry weight.

**Table 7:** Sterol contents in tomato (mg/kg dry wt).

Sterols	Concentrations (mg/kg)	Range	References
Campesterol	147.50 ± 31.13	100.00–180.00	[5,92]
Stigmasterol	387.50 ± 88.71	260.00–510.00	
Stigmastanol	28.25 ± 10.92	10.00–38.00	
β-sitosterol	720.00 ± 175.64	520.00–1000.00	
Δ5-Avenasterol	62.30 ± 2.21	10.00–65.87	
Cholestanol	9.70 ± 1.80	2.10–11.54	
Cholest-7-en-3-ol	3.60 ± 0.13	0.42–4.40	
Cholesterol	41.90 ± 2.10	8.40–43.45	
Lanost-8-en-3-β-ol	52.40 ± 6.80	4.50–60.65	
24-Oxocholesterol	67.50 ± 3.20	14.20–70.69	
Total sterol	1283.25 ± 239.39	918.00–1570.00	

Concentrations are expressed as mean ± standard deviation.

**Table 8:** Antioxidant constituents in tomato (fresh weight).

Antioxidant Constituents	Units	Concentrations	Range	References
α-tocopherol	mg/100 g	0.701 ± 0.110	0.59–0.88	[3,5,49,50,61,65,90,113]
β-tocopherol	mg/100 g	0.030 ± 0.004	0.02–0.03	
γ-tocopherol	mg/100 g	0.810 ± 0.720	0.40–2.24	
δ-tocopherol	mg/100 g	0.020 ± 0.010	0.01–0.02	
Total tocopherol	mg/100 g	1.200 ± 0.150	1.02–1.44	
Vitamin C	mg/100 g	13.990 ± 3.73	9.03–23.80	
β-carotene	mg/100 g	0.420 ± 0.080	0.30–0.51	
Lycopene	mg/100 g	7.960 ± 1.780	5.02–9.49	
Phenolic acids	mg CIAE/g extract	25.500 ± 3.590	21.34–31.23	
Flavonoids	mg QE/g extract	4.230 ± 1.280	3.06–6.36	
Anthocyanins	mg ME/g extract	0.870 ± 0.470	0.23–1.36	

Concentrations are expressed as mean ± standard deviation. CIAE: chlorogenic acid equivalents. QE: quercetin equivalents. ME: malvidin 3-glucoside equivalents.
